# Linking knowledge, attitudes, and practices to sustainable solid waste, water, and energy use in Egyptian households

**DOI:** 10.1038/s41598-025-29799-1

**Published:** 2025-12-23

**Authors:** Alaa K. Ibrahim, Nesma Lotfy, Amira Mahboob, Mohamed Fakhry Hussein

**Affiliations:** 1 Environmental Engineering, Department of Environmental Health, High Institute of Public Health, Alexandria University, 165 El-Horreya Avenue-El-Ibrahimia, Alexandria, Egypt; 2 Biostatistics, Department of Biostatistics, High Institute of Public Health, Alexandria University, 165 El-Horreya Avenue-El-Ibrahimia, Alexandria, Egypt; 3 Environmental Health, Department of Occupational Health and Industrial Medicine, High Institute of Public Health, Alexandria University, 165 El-Horreya Avenue, El-Ibrahimia, Alexandria, Egypt

**Keywords:** Energy conservation, Knowledge, Sustainable practices, Solid waste management, Water conservation, Environmental sciences, Environmental social sciences, Environmental studies, Hydrology

## Abstract

Municipal solid waste management (SWM) and water and energy conservation in Egypt have been the subject of several prior studies. However, less is reported concerning household-level knowledge, attitudes, and behaviors (KAP) in these domains. Therefore, this study aimed to evaluate the Kap of Egyptian households and identify the factors impacting SWM and conservation actions. It utilized an online questionnaire to collect data on KAP regarding SWM, as well as water and energy conservation, from Egyptian households. The results revealed that participants had a high level of knowledge and positive attitudes toward SWM, as well as water and energy conservation. However, their actual practices related to SWM were moderate, while their practices concerning water and energy conservation were relatively low. The results also indicated that gender, age, marital status, and educational level were significantly related to the knowledge of SWM. In contrast, age and education were found to be the only key indicators of knowledge in water and energy conservation. Furthermore, it was found that higher levels of education were associated with higher knowledge, attitudes, and practices (KAP) ratings in SWM as well as water and energy conservation.

**Supplementary Information:**

The online version contains supplementary material available at 10.1038/s41598-025-29799-1.

## Introduction

Achieving sustainable development has been a challenge since the beginning of the 21st Century. The UN’s Sustainable Development Goals (SDGs) report many sustainability issues, especially SDG 12, which urges shifts toward sustainable consumption and production^1^. Household consumption is vital as consumers’ decisions influence sustainability practices^2^.

Developing nations, particularly those with warmer climates like Egypt, are more susceptible to climate change. Many studies explored how climate change is affecting energy production and water resources^3^, emphasizing the need for significant shifts in consumer behavior around waste management, energy, and water.

With solid waste generation rising along with the world’s population, it is becoming a pressing concern^4^. About 26 million tons of municipal solid waste are generated in Egypt yearly, with each person generating between 0.3 and 2.0 kg/day. This includes about 56% organics, 13% plastics, 10% paper and cardboard, 4% glass, and 2% metals^5^. Currently, 81% are openly dumped, 7% are sent to landfills, and only 12% are recycled. The Egyptian Waste Management Regulatory Authority (WMRA) aims to enhance the recycling and composting efforts^6^. Since recycling is considered a sustainable waste management technique, effective programs should focus on raising societal awareness and attitudes to improve recycling behaviors^7^.

As water shortage became a global issue, sustainable water programs became a must. In Egypt, the population reached 101.9 million in 2021, increasing the need for new settlements in the desert. Egypt’s annual water consumption is about 114 billion cubic meters (BCM), but only 60 BCM are accessible. Supplying fresh water to these settlements is costly due to their distance from the Nile River, high elevation, and residents’ high water consumption rates. To tackle this issue, it is essential to manage existing resources more efficiently^8^. Hence, public awareness is crucial for successful initiatives, as users’ behavior significantly impacts water consumption, which indicates a need for more research focused on user knowledge, attitude, and behavior^9^.

Regarding energy conservation, governments are prioritizing it as a key sustainability issue because of its impact on global warming^10^. Research indicated that simply investing in technology does not guarantee low energy consumption; understanding human attitudes and behaviors is essential^11^. In Egypt, the residential sector accounts for 42.4% of total electricity consumption, with excessive use of air conditioning in the cooling season being the primary factor. Energy demand is predicted to rise due to rapid urbanization and population growth^12^. Implementing Demand Side Management (DSM) techniques can shift consumption to off-peak periods and decrease total electricity bills^13^. However, there is currently a lack of understanding of households’ preferences for energy conservation, highlighting the need to study consumer behaviors, as well as the barriers and incentives involved.

Although earlier studies revealed a worldwide gap in awareness of sustainable consumption, this research offers two new findings: first, it shows how material conditions restrict eco-friendly practices by combining the survey and appliance-level data (such as age and usage frequency); second, it employs path analysis to measure how attitudes control knowledge-behavior gaps. This study could aid policymakers and local authorities in creating effective awareness campaigns and sustainability strategies, as it aims to measure the KAP of Egyptian households concerning two critical areas of sustainability: SWM and Water and Energy Conservation, and also identify the socio-demographic determinants (such as age, gender, education, and marital status) that influence the observed KAP scores among the participants.

## Methodology

A cross-sectional online survey was conducted in Egypt between November 16, 2023, and September 15, 2024. Participants were eligible if they were adult Egyptians aged 18 or older, had at least 1 year of residence in their current home, and had internet access via computer or smartphone.

### Sample size and method of selection

Supposing that 50% of the participants have good knowledge about environmental sustainability, the margin of error is 5%, the confidence interval is 95%, and the minimum required sample size is 385. The sample size was calculated using Epi-Info software. The desired sample size was gathered using a non-random sampling design (convenience and snowball sampling techniques). Social media websites such as Facebook, WhatsApp, Telegram, and Messenger were used for the distribution of the questionnaire.

The survey was conducted across the country, with participants from several Egyptian governorates. However, due to the online convenience sampling process, distribution across all geographical regions may not be perfectly balanced.

### Data collection tools

An anonymous self-administered structured questionnaire, as demonstrated in Appendix 1, was formulated based on previously validated questionnaires and after extensive review of previous literature^14–16^. To ensure the quality of the questionnaire, the content validity and reliability were assessed. Content validity was established through expert review by a committee of professors in public health, sustainability, civil engineering, and environmental health. The committee ensured the relevance, clarity, and comprehensiveness of the questionnaire designed to measure the intended construction. A pilot study was conducted with 20 participants to evaluate the practicality and efficacy of the research instrument and data collection methods. The questionnaire took approximately 10–15 min to complete, and minor adjustments were made to improve clarity and flow.

The reliability of the questionnaire was assessed using Cronbach’s alpha. The Cronbach’s alpha values were as follows: 0.667 for the solid waste management knowledge, 0.617 for the solid waste management attitude, 0.676 for the knowledge section on water and energy-saving concepts, and 0.834 for the attitude section on water and energy-saving methods. Cronbach’s alpha above 0.6 indicates an acceptable reliability^17^.

The predesigned questionnaire was uploaded to a Google Form. Participants could choose to complete the survey in Arabic or English. [Table 1] represents the main content of each section of the questionnaire and the way of scoring.

**Table 1:** The main content of the questionnaire and its scoring.

Section	Main content	Scoring
1. Socio-demographic information	Age, sex, nationality, residence, marital status, education level, occupation, income, family size	Not applicable
2. Knowledge about SWM	Definition and hazards of unsafe solid waste disposal	Correct: 1, Incorrect/I do not know: 0; Total: 0–7
3. Attitudes towards SWM	Opinions on solid waste reduction methods	5-point Likert scale (5 to 1); Total: 5–35
4. Practices regarding SWM	Collection and disposal methods	Desired: 3, Undesired: 1; Total: 2–8
5. Knowledge of water & energy-saving concepts	Understanding of water and energy-saving concepts	Correct: 1, Incorrect/I do not know: 0; Total: 0–8
6. Attitudes towards water & energy-saving methods	Opinions on home conservation methods	5-point Likert scale (5 to 1); Total: 5–45
7. Water and energy-saving practices	Behaviors related to conservation	Desired: 1, Undesired: 0; Total: 1–8
8. Energy consumption	Number, age, and frequency of use of electrical devices	Not applicable

### Ethical considerations

The researchers obtained approval from the Ethics Committee of the High Institute of Public Health, Alexandria University, Egypt (IRB number: 00013692, serial number: AU0923926382, date of approval: 26/9/2023), and adhered to the International Guidelines for Research Ethics laid down in the 1964 Declaration of Helsinki and its later amendments. Participants were informed of the research purpose at the beginning of the questionnaire, and each answered a question to confirm their acceptance of participation. The answer of “yes” was considered consent to participate in the study. Participants were able to withdraw from the survey at any time. Anonymity and confidentiality were assured and maintained.

### Statistical analysis

Baseline characteristics were described using means and standard deviations for continuous measures; counts and percentages were used for categorical measures. An independent t-test was conducted to compare the means between two different groups, while an ANOVA test was used for more than two groups. Pearson correlations were studied to evaluate the correlation between participants’ knowledge, attitude, and practice. The analysis was done using IBM SPSS-25 (Statistical Package for the Social Sciences).

A path analysis model was conducted to examine the relationships among variables using SPSS-AMOS. Firstly, a full model was fitted, incorporating all the relationships between variables identified in the univariate analyses. Then, a reduced model was created by removing the relationships that were not significant in the whole model. Only the reduced model is presented. Bias-corrected confidence intervals were calculated to determine the significance of the indirect effect. The indirect effect ratio was computed in consistent mediation as the indirect effect divided by the total effect. Goodness-of-fit statistics, such as root mean square error approximation (RMSEA) (RMSEA < 0.05), chi-square, comparative fit index (CFI) (CFI > 0.95), goodness-of-fit index (GFI) (GFI > 0.95), and adjusted goodness-of-fit index (AGFI) (AGFI > 0.95), were used to evaluate the model fit^18^. All statistical tests were two-tailed and performed at a 5% significance level.

## Results

### Sociodemographic data

According to the findings, most of the participants (61%) were female, as shown in (Table 2). 45% were in the 31–40 age group, 26.5% aged 21–30, and only a small proportion (6.5%) were 51 years or older. The table also showed that most of the participants (59%) were married. Almost 40% had completed postgraduate education, and 38% had completed university education. A significant proportion of participants (61.36%) came from small families (1–4 members). The occupation distribution displayed a relatively diverse set of roles, with the largest group (23%) in the medical field, while 9% were not working.

**Table 2:** Sociodemographic characteristics of the study participants.

Variables	*N*(%) *N* = 396
Gender	
Male	155 (39)
Female	241 (61)
Age	
18–20	36 (9.5)
21–30	105 (26.5)
31–40	179 (45)
41–50	50 (12.5)
51+	26 (6.5)
Marital status	
Single	163 (41)
Married	233 (59)
Highest level of education	
Before university	90 (23.0)
University	149 (37.5)
Postgraduate	157 (39.5)
Family size	
1–4	243 (61.36)
5+	153 (38.64)
Occupation	
Teacher	77 (19.4)
Medical field	92 (23.2)
Engineer	31 (7.8)
Accountant	19 (4.9)
Clerk	19 (4.9)
Salesman	24 (6.0)
Student	38 (9.6)
Not working	37 (9.3)
Others	59 (14.9)

### SWM (solid waste management)

#### KAP (knowledge, attitude, practice)

Data from (Table 3) indicated that female participants had significantly higher mean knowledge and practice scores (5.22 ± 1.45) and (4.28 ± 1.07), respectively, compared to male participants (3.97 ± 1.96) and (3.81 ± 1.24), respectively. However, the gender difference in attitude was not statistically significant. The mean knowledge and practice scores varied significantly across age groups, with the youngest age group (18–20) having the lowest mean knowledge and practice scores (3.36 ± 1.78) and (3.72 ± 1.23). There was no statistically significant difference in the mean attitude scores across the different age groups. While being married was significantly associated with a higher mean knowledge score (5.00 ± 1.59) compared to being single (4.34 ± 1.94), the marital status had no statistically significant association with the mean attitude and practice scores. The educational level of the participants showed a statistically significant association with their mean KAP scores. Postgraduates and university students had the highest mean KAP scores compared to individuals with lower educational levels. 

**Table 3:** Knowledge, attitude, and practice of study participants regarding solid waste management.

	Knowledge of SWM Mean (SD)	*p*	Attitude of SWM Mean (SD)	*p*	Practice of SWM Mean (SD)	*p*
Gender						
Male	3.97 (1.96)	0.000	26.99 (3.48)	0.069	3.81 (1.24)	0.000
Female	5.22 (1.45)		27.63 (3.24)		4.28 (1.07)	
Age						
18–20	3.36 (1.78)	0.000	26.92 (2.92)	0.126	3.72 (1.23)	0.001
21–30	4.24 (2.00)		26.87 (3.42)		3.83 (1.09)	
31–40	5.20 (1.49)		27.59 (3.24)		4.23 (1.14)	
41–50	4.96 (1.64)		27.46 (3.82)		4.50 (1.25)	
51+	4.92 (1.47)		28.58 (3.15)		4.04 (0.96)	
Marital status						
Single	4.34 (1.94)	0.000	27.28 (3.43)	0.614	3.96 (1.17)	0.052
Married	5.00 (1.59)		27.45 (3.30)		4.19 (1.14)	
Highest level of education						
Before university	2.92 (1.70)	0.000	25.91 (2.97)	0.000	3.43 (1.07)	0.000
University	4.95 (1.49)		27.50 (3.27)		4.32 (1.13)	
Postgraduate	5.55 (1.26)		28.11 (3.38)		4.27 (1.10)	
Overall score (mean (SD))	4.73 (1.77)		27.38 (3.34)		4.09 (1.15)	

#### Correlation between Knowledge, Attitude, and Practice 

There was a statistically significant moderate positive correlation between participants’ knowledge regarding SWM and their attitude, as evidenced by (Table 4). Furthermore, there was a significant weak positive correlation between knowledge and practice, as well as between participants’ attitude towards SWM and their practice.

**Table 4:** The correlation between Knowledge, attitude, and practice of the study participants regarding solid waste management.

SWM			
	Knowledge	Attitude	Practice
Knowledge	1	0.411 (p<0.001)	0.334 (p<0.001)
Attitude		1	0.293 (p<0.001)
Practice			1

#### Path analysis model

**Fig. 1:**
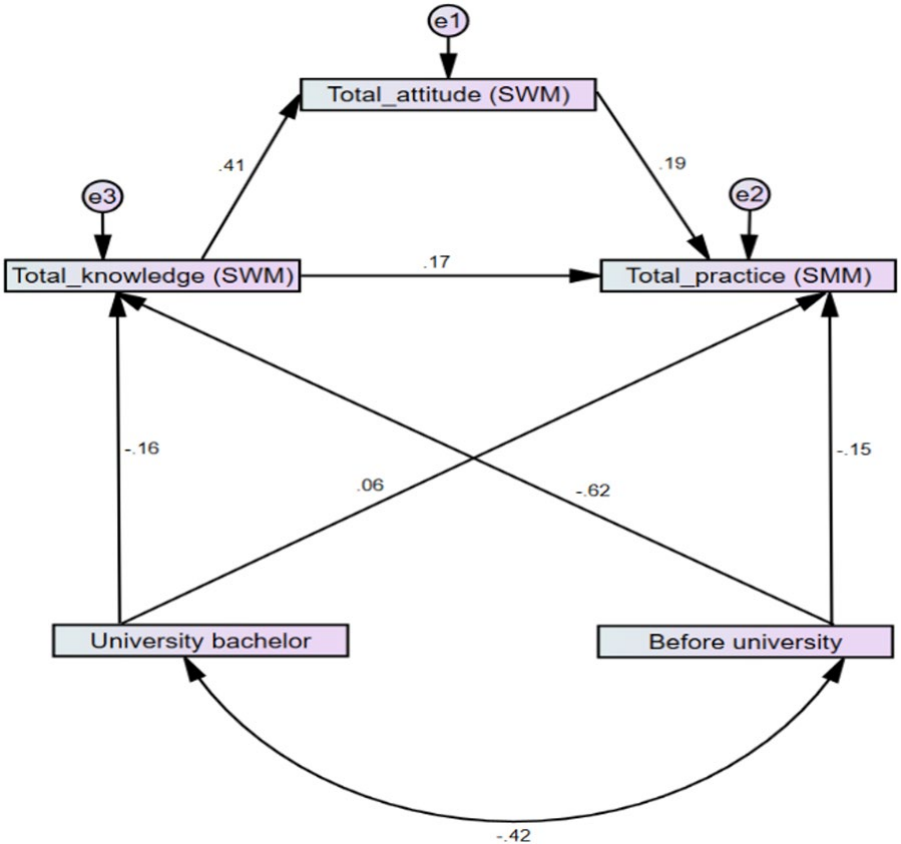
Standardized coefficients of the reduced model for SWM.

The reduced path analysis model for SWM was illustrated in (Fig. 1) and (Table 5). Participants before university and those with a university degree had lower knowledge and practice of SWM than those with higher education (standardized coefficient = − 0.62, *p* < 0.001; standardized coefficient = − 0.15, *p* = 0.016, respectively). A similar pattern was observed among participants with a university degree, who demonstrated lower knowledge of SWM compared to those with higher education (standardized coefficient = − 0.16, *p* < 0.001). The model revealed that an increase in knowledge of SWM led to an increase in attitude, which, in turn, increased practice. Additionally, there was a statistically significant indirect effect of knowledge on practice through attitude (indirect standardized effect coefficient = 0.076, *p* = 0.011; indirect effect ratio = 30.89%). Furthermore, pre-university status had a statistically significant indirect effect on practice through knowledge and attitude (indirect standardized effect coefficient = − 0.153, *p* = 0.014; indirect effect ratio = 50.8%). The model showed good model fit, i.e., RMSEA = 0.000, GFI = 1, AGFI = 0.998, and CFI = 0.998.

**Table 5:** Model parameters and fitting of SWM.

SWM model				
Variable 1		Variable 2	Std.coeff.	p-value
Before university	→	Knowledge SWM	− 0.62	0.000
University bachelor	→	Knowledge SWM	− 0.16	0.000
Before university	→	Practice SWM	− 0.15	0.016
University bachelor	→	Practice SWM	0.06	0.230
Knowledge SWM	→	Attitude SWM	0.41	0.000
Attitude SWM	→	Practice SWM	0.19	0.000
Knowledge SWM	→	Practice SWM	0.17	0.005
Model fit				
Chi-square (p-value)			0.297(0.862)	
CFI			1	
GFI-AGFI			1 − 0.998	
RMSEA			0.000	
Indirect effect (indirect standardized effect coefficient, p-value, and indirect effect ratio)				
Before university → Practice SWM			− 0.153 (0.014) (50.8%)	
University bachelor → Practice SWM			− 0.04(0.009) (not calculated)	
Knowledge SWM → Practice SWM			0.076 (0.011) (30.89%)	

### Water and energy conservation

#### KAP (knowledge, attitude, practice)

Gender differences in knowledge and attitude regarding water and energy conservation, as highlighted in (Table 6), were statistically significant, with females having higher mean scores (5.92 ± 1.39) and (39.16 ± 3.44) than males (4.90 ± 2.15) and (35.90 ± 5.63). However, there was no statistically significant difference in the mean practice scores between males and females. Additionally, the data showed that age, marital status, and education level all had significant associations with the mean KAP scores. Older individuals, married individuals, and those with higher levels of education demonstrated better knowledge, attitude, and practice. Nevertheless, the association between marital status and the mean knowledge scores was not statistically significant.

**Table 6:** Knowledge, attitude, and practice of study participants regarding water and energy conservation.

	Knowledge of water and energy conservation Mean (SD)	*P*	Attitude of water and energy conservation Mean (SD)	*P*	Practice of water and energy conservation Mean (SD)	*P*
Gender						
Male	4.90 (2.15)	0.000	35.90 (5.63)	0.000	3.92 (1.45)	0.558
Female	5.92 (1.39)		39.16 (3.44)		3.83 (1.45)	
Age						
18–20	5.03 (1.81)	0.000	35.92 (4.86)	0.000	3.39 (1.20)	0.013
21–30	4.55 (2.12)		35.18 (5.57)		3.58 (1.32)	
31–40	4.95 (1.44)		39.29 (3.67)		4.08 (1.54)	
41–50	6.18 (1.37)		39.08 (3.50)		3.93 (1.33)	
51+	5.92 (1.62)		39.54 (3.26)		4.10 (1.56)	
Marital status						
Single	5.31 (1.96)	0.052	37.25 (5.19)	0.03	3.62 (1.38)	0.006
Married	5.67 (1.66)		38.33 (4.28)		4.03 (1.48)	
Highest level of education						
Before univ	3.80 (2.13)	0.000	32.63 (4.83)	0.000	3.22 (1.03)	0.000
University	5.84 (1.47)		38.68 (3.58)		3.89 (1.40)	
Postgraduate	6.21 (1.12)		40.14 (2.98)		4.21 (1.59)	
Overall score (mean (SD))	5.5 (1.7)		37.88 (4.7)		3.8 (1.4)	

#### Correlation between knowledge, attitude, and practice

A statistically significant moderate positive correlation was found between participants’ knowledge of water and energy conservation and their attitude, as well as a significant weak positive correlation between knowledge and practice (Table 7). Moreover, there was also a significant weak positive correlation between participants’ attitude towards water and energy conservation and their practice.

**Table 7:** The correlation between knowledge, attitude, and practice of the study participants regarding water and energy saving.

Water and energy conservation			
	Knowledge	Attitude	Practice
Knowledge	1	0.659 (0.000)	0.278 (0.000)
Attitude		1	0.301 (0.000)
Practice			1

#### Path analysis model

**Fig. 2:**
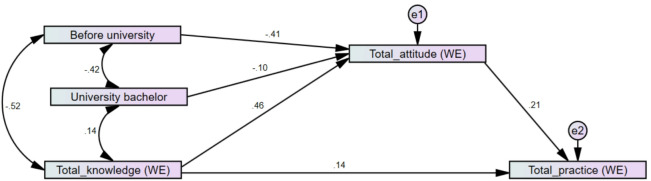
Standardized coefficients of the reduced model for water and energy (WE) conservation.

Participants before university and those with a university degree had lower attitudes toward water and energy conservation compared to those with higher education (standardized coefficient = − 0.41, *p* < 0.001; standardized coefficient = − 0.10, *p* = 0.006, respectively). This was illustrated by the reduced path analysis model for water and energy conservation, as demonstrated in Fig. 2 and Table 8. The model revealed that an increase in knowledge of water and energy conservation led to an increase in attitude, which, in turn, increased practice. Additionally, there was a statistically significant indirect effect of knowledge on practice through attitude (indirect standardized effect coefficient = 0.095, *p* = 0.012; indirect effect ratio = 40.08%), of pre-university status on practice through attitude (indirect standardized effect coefficient = -0.085, *p* = 0.016; indirect effect ratio = 100%), and of university degree status on practice through attitude (indirect standardized effect coefficient = − 0.022, *p* = 0.005; indirect effect ratio = 100%). The model showed good model-fit, i.e., RMSEA = 0.034, GFI = 0.997, AGFI = 0.978, and CFI = 0.998.

**Table 8:** Model parameters and fitting of water and energy conservation.

WE model				
Variable 1		Variable 2	Std. coeff.	p-value
Before university	→	Attitude WE	− 0.41	0.000
University bachelor	→	Attitude WE	− 0.10	0.006
Knowledge WE	→	Attitude WE	0.46	0.000
Attitude WE	→	Practice WE	0.21	0.001
Knowledge WE	→	Practice WE	0.14	0.025
Model fit				
Chi-square (p-value)			2.938 (0.23)	
CFI			0.998	
GFI – AGFI			0.997–0.978	
RMSEA			0.034	
Indirect effect (indirect standardized effect coefficient, p-value, and indirect effect ratio)				
Before university → Practice			− 0.085 (0.016) (100%)	
University bachelor → Practice			− 0.022 (0.005) (100%)	
Knowledge → Practice			0.95 (0.012) (40.08%)	

### Home appliance practice

**Fig. 3:**
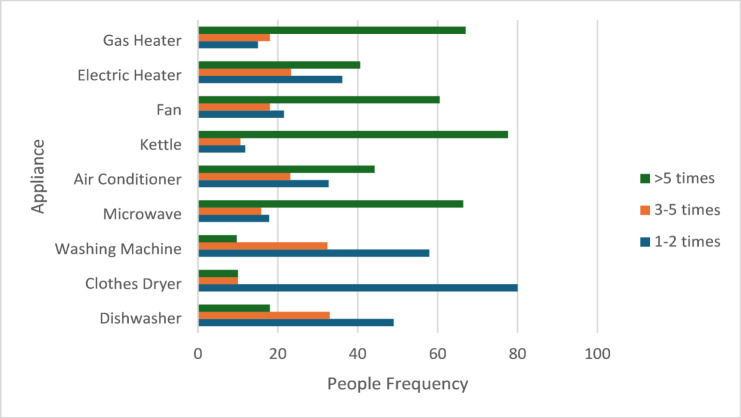
Appliance frequency of usage per week.

The variety of home appliances owned by the study population was displayed in Table 9 and Fig. 3. Household ownership of essential items was very high: 93.4% owned a washing machine, 96.2% had a television, and 94.2% had a gas heater. Conversely, only 10.1% of families owned an energy-intensive non-essential device, such as a clothes dryer, while 23.7% owned a dishwasher.

**Table 9:** Home appliance categories, numbers, and age.

Device	Number of owned appliances *N* (%)			Age of the appliance *N* (%)*			
	0	1	2+	< 2 years	2–5 years	> 5 years	don’t know
Dishwasher	302(76.3)	92(23.2)	2(0.5)	12(12.8)	31(32.9)	42(44.7)	9(9.6)
Clothes dryer	356(89.9)	37(9.3)	3(0.8)	1(2.5)	16(40)	12(30)	11(27.5)
Washing machine	26(6.6)	346(87.7)	24(6.1)	8(2.2)	145(39.2)	175(47.3)	42(11.3)
Microwave	194(49)	199(50.3)	3(0.8)	6(3)	80(39.6)	101(50)	15(7.4)
Large fridge (more than 600 L	265(66.9)	121(30.6)	10(2.5)	4(3.1)	55(41.9)	59(45)	13(10)
Medium fridge (300–600 L)	152(38.4)	235(59.3)	9(2.3)	7(2.9)	79(32.3)	125(51.2)	33(13.6)
Small fridge (less than 300 L)	327(82.6)	65(16.4)	4(1.0)	---	30(43.5)	20(29)	19(27.5)
Air Conditioner	188(47.5)	111(28)	97(24.5)	12(5.8)	80(38.5)	99(47.5)	17(8.2)
Television	15(3.8)	249(62.9)	132(33.3)	12(3.2)	150(39.4)	178(46.7)	41(10.7)
Kettle	142(35.9)	240(60.6)	14(3.5)	19(7.5)	86(33.9)	119(46.8)	30(11.8)
Computer/Laptop	85(21.5)	207(52.3)	104(26.3)	9(2.9)	100(32.2)	172(55.3)	30(9.6)
Fan	24(6.1)	148(37.4)	224(56.6)	10(2.7)	131(35.2)	195(52.4)	36(9.7)
Electric heater	263(66.4)	112(28.3)	21(15.8)	9(6.8)	49(36.8)	52(39.1)	23(17.3)
Gas heater	129(32.6)	253(63.9)	14(3.5)	4(1.5)	103(38.6)	126(47.2)	34(12.7)

*The denominator is the total number of participants who reported having the appliance.

A large percentage of home appliances were older than five years. About 47.5% of the seven most common appliances (Televisions, Fans, Washing Machines, Computers/Laptops, Gas heaters, kettles, and microwaves) were older than five years. Regarding refrigerators, Results showed that most households preferred medium-sized refrigerators (300–600 L), with 59.3% owning one. Furthermore, more than 51% of medium refrigerators and 45% of large refrigerators are older than 5 years.

In addition, a knowledge gap across all devices was found, as more than 27.5% of households could not remember the age of their appliances. This was mainly noticeable for less common devices (Electric heaters, Clothes dryers, and Dishwashers), as 17.3% of owners did not know the actual age of these devices.

## Discussion

Since 2000, Egypt has recorded an annual growth in municipal solid waste exceeding 36%. However, Egyptian authorities have not conducted studies on household waste management awareness or practices^19^. Furthermore, with a predicted population of 160 million by 2050, water availability is expected to decrease to less than 350 m^2^ per capita per year^20^. It was also noticed that, since 2001, frequent power interruptions have been a continuous problem^21^. Therefore, it is critical to improve public knowledge about conservation and sustainable practices.

### KAP of participants

#### SWM

In Egypt, KAP of SWM was studied among municipal solid waste workers, health workers, and students, staff, and employees of educational institutes^22–24^. Whereas this study is just limited to households. Municipal SWM in Egypt is considered a significant challenge due to rapid population growth, low awareness, high illiteracy rates, unregulated slum areas, inadequate waste collection and disposal systems, and limited financial resources^25^.

Based on the results, it was found that the overall mean scores of the participants’ knowledge, attitude, and practice for SWM were (4.73 ± 1.77), (27.38 ± 3.34), and (4.09 ± 1.15) out of 7, 35, and 8, respectively, as displayed in [Table 3]. This reveals that participants had a high knowledge and attitude toward municipal SWM. However, their practices were moderate.

Contrary to this study’s results, it was stated that rural women in the rural villages of El-Gharbia Governorate had poor knowledge and practice regarding SWM, yet they had a positive attitude^26^. This might be due to the lack of awareness regarding improper solid waste disposal and management, environmental impact, and the lack of awareness campaigns in Egyptian rural areas. At one of the Sohag governorate villages in Egypt, the families’ knowledge, attitudes, and practices regarding household waste management improved after the implementation of a health awareness package^25^.

#### Water and energy conservation

Regarding water and energy conservation KAP, as displayed in Table 6, the overall mean scores were (5.5 ± 1.7), (37.88 ± 4.7), and (3.8 ± 1.4) out of 8, 45, and 8, respectively. Participants showed high Knowledge and attitude, but low practice levels. This agreed with the results of a study indicating that the UK had positive knowledge and attitudes towards water conservation, but showed poor practices^9^. The same results were found in Spring City, China^27^. Likewise, the people of Gaza had good water and energy conservation awareness and attitudes in their houses^28^.

In a survey conducted in four governorates in Egypt (Cairo, El-Minya, Assiut, and Sohag), a strong correlation was found between participants’ knowledge of the water crisis and their readiness to implement water conservation techniques^8^.

As for energy saving in Egypt, there was a lack of data related to household KAP. Sustainable consumption was only studied among university students^29,30^.

### Effect of gender

#### SWM

According to Table 3, there was a statistical significance between female participants and their knowledge and practices regarding solid waste management. They had higher mean SWM knowledge and practice than males. As per a survey conducted in 2005, it was reported that gender can influence domestic SWM perceptions^7^.

In Egypt, women are seen as the primary generators of municipal SW because they are known to be preoccupied with all household tasks, including preparing food, shopping, and housekeeping, which involves managing and disposing of SW^26^. On the other hand, working females are more dependent on readymade products, leading to increased solid waste^31^. This aligns with the results of a study in Sohag, Egypt, where more than two-thirds of waste managers at the household level were females^25^. Furthermore, another study found that young Egyptian females have good knowledge regarding the use and hazards of plastic bag disposal, while older females over 40 had good practice^32^.

#### Water and energy conservation

Gender norms directly impact the public’s beliefs regarding expectations of home duties^33^. In this study, a statistically significant difference in KAP between genders was revealed; females showed better knowledge and attitude than males, as demonstrated in Table 6. The results were the same as in the two studies that analyzed the KAP of individuals regarding water and energy conservation^34,35^. This also agrees with the findings of a study conducted among university students in Egypt, which found that female students showed better knowledge and practice than male students^29^.

On the other hand, the findings contradicted a study conducted in 2024, which reported that females in Egypt had a lower level of awareness regarding the water crisis^8^. The contradiction may be due to the variation in how awareness and knowledge terms were defined and measured. Also, the geographical scope of the two studies may offer a key explanation for the contradiction.

### Effect of age

#### SWM

The statistical analysis, in Table 3, showed that age had a significant effect on both knowledge (*p* < 0.001) and practice (*p* = 0.001) in WM. Nevertheless, there were no significant differences in attitude within different age groups (*p* = 0.126). The (31–40) age group had the highest knowledge level (5.20 ± 1.49), while the (41–50) age group exhibited the best practices (4.50 ± 1.25). The (18–20) age group had the lowest knowledge and practice levels (3.36 ± 1.78) and (3.72 ± 1.23), respectively. This almost complies with a study conducted in Palestine, where the knowledge and practice levels were much higher in the age group (25–44) than those aged (18–24)^36^. It also aligns with the findings of a study conducted in Egypt, which focused solely on the disposal of plastic bags. The young age group < 40 demonstrated high knowledge, while people aged > 40 exhibited the best practice^32^.

However, another study that specifically focused on waste segregation as a type of SWM practice concluded that respondents aged between 50 and 65 segregated more often than those aged between 35 and 49^37^.

In Egypt, middle-aged groups balance family duties with perceptions gained from education and media, while younger individuals lack maturity due to their limited real-world experiences. In contrast, older generations tend to exhibit practices shaped by their accumulated habits and responsibilities. This is consistent with the Theory of Planned Behavior, which implies that age has an impact on the perceived behavioral control through experience^38^.

#### Water and energy conservation

A one-way ANOVA, as demonstrated in Table 6, revealed statistically significant differences in knowledge, attitude, and practice in terms of water and energy conservation over different age groups. The (41–50) age group had the highest knowledge score (6.18 ± 1.37), while the (18–20) age group had the lowest practice score (3.39 ± 1.20). On the other hand, the 51 + age group had the highest attitude, with a score of (39.54 ± 3.26). Local socio-economic and cultural factors can explain the findings in the Egyptian setting. People of the age (41–50) possibly have the highest knowledge because of their role as household decision makers. On the other hand, the low practice level at the age of 18–20 may reflect their limited responsibilities for utility bills, while a positive attitude in those over 51 may be due to the traditional values of economy in Egyptian culture. However, this positive attitude may not lead to good practice if they lack access to modern water-saving technologies and modern appliances.

These results agree with prior studies showing that elderly individuals conserve water more frequently^39,40^. Furthermore, it was confirmed in another study that people aged 35 will consume 26% more energy than people aged 70^41^. This could be due to the observation that older people have fewer home appliances than younger ones, which leads to a decrease in energy consumption^42^.

### Effect of marital status

#### SWM

The results, displayed in Table 3, show a significant difference in knowledge scores between single and married individuals (*p* < 0.001). Those who were married (5.00 ± 1.59) had much higher knowledge levels than single participants. Meanwhile, no significant variations were found between the groups regarding their attitudes and practices towards SWM.

This is in agreement with the results of two studies conducted in 2021 and 2022, which found that marital status is non-significant in household solid waste practice^37,43^. At the same time, two different studies discovered an association between marital status and improper solid waste management^44,45^.

In Egypt, marriage indicates moving to an independent household, leading couples to engage in daily SWM. However, there were no significant differences in attitudes and practices, which means that while marriage can aid in gaining knowledge, these attitudes and practices might be hindered. This is due to poor recycling infrastructure and inconsistent waste collection.

#### Water and energy conservation

The analysis from [Table 6] stated that married participants reported better practices (*p* = 0.006) compared to single individuals. However, no significant difference in knowledge levels was observed between the two groups (*p* = 0.052).

Married people adopted more energy-saving behaviors than the others^46^. This can be attributed to the economic pressure that marriage causes in Egypt, as it exposes married couples to the financial cost of utility bills. This creates motivation towards water and energy conservation. On the other hand, it was concluded in another study that married people tend to save less water than singles^39^. The contradiction may be due to cultural differences in water availability, utility bills, and housing situations.

### Effect of education

The path analysis model, shown in Figs. 1 and 2 and stated in Tables 5 and 8, proved that higher education levels improve the knowledge level of SWM as well as water & energy conservation. This, in turn, will enhance attitudes and sustainable practices. The results indicated that education level was the most important factor affecting participants’ KAP.

#### SWM

The results highlighted a significant association between the education level and solid waste management KAP (*p* < 0.001). Moreover, a pattern was discovered, as mean scores improved from the Before university to the University and Postgraduate groups. [Table 3] showed that postgraduates and university students have higher mean KAP scores for SWM, (4.95 ± 1.49) & (5.55 ± 1.26) respectively, than those with lower educational levels.

Education levels had an impact on the awareness, attitude, and practices of SWM^47,48^. Nowadays, the higher education system in Egypt includes more topics regarding environmental science and sustainability, which equip students with essential knowledge^49^. Being skilled in English and digital literacy also allowed them to better understand global sustainability issues.

#### Water and energy conservation

It was also noted that an increase in education level leads to an enhancement in the KAP of water and energy conservation, based on the results from [Table 6]. Several studies support the belief that households with high education levels frequently have stronger intentions toward water or energy conservation^40,50,51^.

A significant observation in this study’s results was that improved knowledge of water and energy conservation promotes positive attitudes, leading to enhanced practices. The model showed that knowledge does not automatically result in action. Instead, it creates a favorable mindset and a sense of responsibility. This strengthened attitude serves as the crucial step, eventually motivating individuals to adopt sustainable behaviors in their daily lives.

Financial challenges and rising utility bills often increase households’ need for practical and cost-effective approaches. Families tend to actively seek advice on reducing consumption, such as understanding appliance energy use, taking shorter showers, and using appliances more efficiently. This knowledge becomes essential for financial survival. However, the stress of managing limited finances can hinder engagement with broader information campaigns and education focused on long-term environmental benefits. High initial costs of energy-efficient appliances or water-saving technologies can make them seem irrelevant. Thus, while financial strain may raise interest in immediate, low-cost savings, it can limit curiosity about knowledge that requires financial commitment or does not result in quick bill reductions.

In Egypt, water scarcity is a pressing issue that strengthens the connection between attitude and action^52^. Due to limited resources and economic pressure, education is considered vital for providing individuals with the knowledge needed to mitigate sustainability challenges^49^.

### Home appliances practice

Around 47% stated that their home appliances were more than 5 years old, as shown in Table 9. Older appliances may have lower energy efficiency compared to contemporary models^53^, and this raises a conflict between environmental regulations and residential energy use. On one side, keeping these appliances may increase energy consumption and greenhouse gas emissions. On the other hand, appliance replacement would result in a significant amount of e-waste. The recycling and management of e-waste have a considerable environmental impact^54^. As a result, a trade-off exists between enhancing energy efficiency and addressing the environmental effects of appliance manufacturing and disposal. This is further complicated by the study results, which indicate a knowledge gap. Many respondents were unaware of their appliances’ age, indicating a potential lack of awareness about long-term energy costs and the environmental impacts associated with appliance ownership.

The long lifespan of home appliances confuses consumers as to whether to replace or fix their devices, and each phase of the home appliance life cycle affects the environment. The correlation between life span, repair frequency, and maintenance behavior was assessed, and it was suggested that the environmental effects of early replacement for household devices might differ based on numerous factors, including the appliance type, the new model’s energy efficiency, and the methods employed for disposing of the old appliance^55,56^.

The most reported devices used in the week, as presented in Fig. 3, were kettles, gas heaters, microwaves, and fans, with percentages of 77.6%, 67%, 66.4%, and 60.5%, respectively. The survey did not include fridges, TVs, computers, or laptops in this question, as it was assumed that these are the most used devices in every house. For example, fridges work nonstop the whole day. Considering the climate conditions and culture, the observed usage frequencies make sense. However, one of the main factors influencing energy demand is the significant use of air conditioners, particularly by households that own them (24.5% have two or more units). The number of home appliances has increased over the last 20 years. The frequency of using these appliances plays a significant role in energy consumption^57^. About 70% of houses’ carbon dioxide emissions are generated from home appliances. Air conditioners, refrigerators, and televisions are responsible for half of those carbon dioxide emissions^58^. Other studies revealed that unreasonable usage and purchasing habits of home appliances were the leading causes of energy waste in houses^59^. According to the Egyptian Electricity Holding Company, Domestic energy consumption was responsible for more than half of the total energy consumption in Egypt^60^.

### Study limitations

The current study has several limitations. The non-randomized sampling and reliance on an online questionnaire likely introduced selection bias, favoring those with internet access and digital literacy. Accordingly, there was an overrepresentation of highly educated participants compared to the national average, and the sample did not fully represent all Egyptian governorates, limiting the generalizability of the findings. In addition, self-reported data may have introduced biases, such as recall or social desirability bias. Although the study measured attitudes and behaviors effectively, it may have failed to detect the value-action gap that arises from external barriers such as financial constraints.

## Conclusion

This study successfully measured the Knowledge, Attitudes, and Practices (KAP) of Egyptian households toward SWM and Water and Energy Conservation and identified key socio-demographic determinants. It was found that participants had a high level of knowledge and positive attitudes toward SWM, as well as water and energy conservation. However, their actual practices related to SWM were moderate, while their practices concerning water and energy conservation were relatively low.

It was also found that female participants exhibited higher levels of knowledge and practices for SWM and greater knowledge and attitudes for water and energy conservation compared to males. Regarding SWM, the younger age groups demonstrated lower KAP scores. For water and energy conservation, older, married, and highly educated individuals reported better overall KAP. The data indicates a clear pathway: a rise in knowledge directly leads to a more positive attitude, which in turn results in improved sustainable practices for both SWM and water/energy conservation. These results confirm the critical role of demographic factors and knowledge-attitude pathways in shaping sustainable practices within Egyptian households.

## Supplementary Information

Below is the link to the electronic supplementary material.

Supplementary Material 1

## Data Availability

All data generated or analyzed during this study are included in this article and are available from the corresponding author upon reasonable request.
