# Supplementary material for: Linking knowledge, attitudes, and practices to sustainable solid waste, water, and energy use in Egyptian households
Source: Sci Rep. 2025 Dec 23;15:44409. doi: 10.1038/s41598-025-29799-1 (PMC12739171; doi:10.1038/s41598-025-29799-1)
Supplement: Supplementary file 1 — Supplementary Material 1 [file 41598_2025_29799_MOESM1_ESM.pdf]

Linking Knowledge, Attitudes, and Practices to Sustainable Solid Waste, Water, and Energy Use in Egyptian Households

Alaa K. Ibrahim [a*], Nesma Lotfy [b], Amira Mahboob [c], Mohamed Fakhry Hussein [c]

[a*] *Environmental Engineering, Department of Environmental Health, High Institute of Public Health, 165 El-Horreya Avenue – El-Ibrahimia, Alexandria University, Alexandria, Egypt. ORCID:0000-0002-1302-8788*

[b] *Biostatistics, Department of Biostatistics, High Institute of Public Health, 165 El-Horreya Avenue – El-Ibrahimia, Alexandria University, Alexandria, Egypt*

[c] *Environmental Health, Department of Occupational Health and Industrial Medicine, High Institute of Public Health, 165 El-Horreya Avenue, El-Ibrahimia, Alexandria University, Alexandria, Egypt*

I) Personal and Demographic information:

1. Governorate: ……………….

2. Gender: ……………………..

3. Age: ………………………..

4. Marital status:

- Single
- Married
- Widowed
- Divorced

5. Highest level of education you completed:

- Primary Education
- Secondary Education
- Vocational and Technical Education
- University Bachelor
- Postgraduate Studies

6. Occupation: ………………………

II) Part A: Solid Waste Management (SWM):

a. Knowledge:

	Yes	I don't know	No
Solid Waste is anything without value.			
Solid waste can be sorted and sold to recycling companies.			
Compost or organic fertilizer can be prepared from solid waste.			
The amount of solid waste can be reduced by reusing it at the household level.			
Illegal dumping of solid waste causes diarrhea, typhoid, and cholera.			
Separating different types of solid waste at home helps for Solid Waste Management.			
Improper dumping of solid waste can eventually lead to pollution of rivers, lakes, and wells.			

B. Attitudes (What do you think about the following statements?)

	Strongly agree	Agree	Uncertain	Disagree	Strongly Disagree
Solid waste is one of the environmental problems that needs immediate attention.					
Solid waste can be reduced, reused, and recycled.					
Every household should have responsibility for the proper collection and disposal of solid waste.					
Proper SWM is important for creating a healthy environment.					
Solid Waste Management is a vital issue in my town.					
The government should not make strict and regular supervision and control of illegal dumping of solid waste in the town.					
Selling plastic waste for recycling is the best way to manage solid waste.					

C. Practices:

	Always	Sometimes	Rarely
Do you separate solid waste before disposal?			
Do you reduce, reuse, and/or recycle your solid waste?			

3. How do you get rid of solid waste from home?

- Dumped in the backyard with sacs.
- Dumped along roadsides.
- Dumped in the street containers.
- One person collects waste sacs from my home.

II) Part B: Water & Energy:

a. Knowledge:

	Yes	I don't know	No
Sustainability is known as "meeting the needs of the present without compromising the ability of future generations to meet their own needs."			
Sustainable development requires an integrated approach that takes into consideration environmental concerns along with economic development.			
When humans interfere with nature, this does not affect the environment			
The amount of water available for use is finite.			
Climate change affects the water availability all over the world.			
Water scarcity & energy depletion have both social and economic implications.			
Population growth and urbanization have an impact on water scarcity & energy depletion.			
Climate change can be caused by burning fuels and electricity.			

B. Attitudes (What do you think about the following statements?)

	Strongly agree	Agree	Uncertain	Disagree	Strongly Disagree
Humans have the right to modify the natural environment to suit their needs.					
Water conservation actions by householders can significantly reduce the amount of water used in urban areas.					
What individual residents do in their homes and gardens has consequences for the health of waterways and coastal bays.					
Humans are severely abusing the environment.					
Plants and animals have as much right as humans to exist.					
Every house should have the right to clean and safe water.					
Both householders and authorities are responsible for water and energy conservation.					
I am interested in learning more about water and energy conservation.					
I am interested in doing anything that contributes to reducing global warming.					

C. Practices:

	Yes	No
1. Do you have or are planning to buy any water-saving devices?		
2. Over the past 5 years, have you actively taken steps to reduce your consumption of water and energy?		

3. Which of the following do you have?

- Roof Insulation
- Wall insulation
- Underfloor insulation
- None of the above
- Don't Know

4. Do you know how much energy & water you use at home?

- Yes, I read my energy & water bills and track my monthly usage.
- Roughly. I glance at my monthly usage as I pay the bills.
- No, I pay the bills and don't look at the usage.
- No. I don't pay my bills (someone else does).

5. In which of the following ways do you conserve water where you live?

- Limit shower time
- Turn off the sink while teeth brushing, hand washing, shaving, etc.
- Only wash full loads of laundry
- Other…………………………

6. What are the actions that you have taken to reduce your energy consumption?

- I have installed smart controls at home to optimize temperature & lighting settings.
- I have installed onsite renewables.
- I regularly take the bus, carpool, or ride my bike to work.
- I turn my thermostat (if you have AC) close to 24 C in the winter, and/or in the summer.
- I purchase energy-efficient appliances.
- I use natural ventilation in my house.
- None of the above.
- Other………………………………

7. How many of the following appliances do you have?

	0	1	2	3	4	5
Dishwasher						
Clothes Dryer						
Washing Machine						
Microwave						
Large fridge (more than 600 L)						
Medium fridge (300-600L)						
Small fridge (less than 300 L)						
Air Conditioner						
Television						
Kettle						
Computer/Laptop						
Fan						
Electric Heater						
Gas Heater						

8. How old are these appliances? If more than one type of appliance choose more than one answer

	Don't have	Don't know	Less than 2 years	2-5 years	More than 5 years
Dishwasher					
Clothes Dryer					
Washing Machine					
Microwave					
Large fridge (more than 600 L)					
Medium fridge (300-600 L)					
Small fridge (less than 300 L)					
Air conditioner					
Television					
Kettle					
Computers/ laptops					
Fan					
Electric heater					
Gas Heater					

14. How many times per week do you use the following appliances?

	Don’t have one	1-2 times a week	3-5 times a week	6-7 times a week	More than 7 times
Clothes Dryer					
Washing Machine					
Dish Washer					
Electric Heater					
Gas Heater					
Microwave					
Kettle					
Air Conditioning					
Electric fans					
